# Inversion method of particle size distribution of milk fat based on improved MPGA

**DOI:** 10.3389/fbioe.2022.964057

**Published:** 2022-09-07

**Authors:** Guochao Ding, Zhen Zhou, Yu Wu, Peng Ji

**Affiliations:** ^1^ College of Information & Electrical Engineering, Heilongjiang Bayi Agricultural University, Daqing, China; ^2^ School of Measurement and Communication on Engineering, Harbin University of Science and Technology, Harbin, China; ^3^ College of Horticulture & Landscape Architecture, Heilongjiang Bayi Agricultural University, Daqing, China

**Keywords:** MPGA, regularization, total light scattering method, dependent, particle size distribution

## Abstract

Milk fat’s particle size and distribution not only affect product quality, but also have great impacts on food safety in the economy and society. Based on total light scattering method, this paper has studied the inversion method of particle size distribution under dependent mode condition by combining multi-population genetic algorithm (MPGA) with Tikhonov smooth function. It has minimized the influence from light-absorb medium to improve the inversion accuracy. The approach introduces Tikhonov smooth function and apparent optical parameters to build an objective fitness function and weaken the ill condition of the particle size inversion equation. It also introduces multi-population genetic algorithm to solve the premature convergence of genetic algorithms. The results show that the relative error of the milk fat simulation solution with a nominal diameter is -3.52%, which meets the national standard of ±8% and better than the relative error of -5.01% of the standard genetic algorithm. Thus, the improved MPGA can reconstruct particle size distribution, with a good reliability and stability.

## Introduction

Particle size and distribution not only affect product performance and quality, but also influence the economy and society in the aspects of environmental protection and human health (Cai et al., 2010). In recent years, the demands for online monitoring of particulate matter have increased, especially in environmental testing and food safety. More scholars from global leading institutions focus on particle size inversion methods.

According to Wang Li’s finding, the method of using regularization to build objective function and establish an improved mode searching model showed a good inversion performance. She also proposed using generalized eikonal approximation to effectively calculate the coefficient of light extinction for measuring the particle size (Wang, 2013; Wang and Sun, 2013). Based on angular scattering method, Mroczka et al. measured particle size distribution using regularization least squares algorithm with constraints. Although they obtained a good inversion result, the team failed to reconstruct the bimodal distribution function (Mroczka and Szczuczyński, 2013). Clementi et al. reversed polystyrene latex particles with different shape, size distribution width and diameter range. They used multi-angle dynamic light scattering and capered Tikhonov and Bayesian methods to solve the ill non-linear problem. The paper concluded that Bayesian method produced better results than Tikhonov regularization (Igushi and Yoshida, 2011). Compared with classical methods, intelligent optimization algorithm showed better whole search ability in measuring particle size distribution and can efficiently overcome the effects from the noise. However, one algorithm cannot have all the advantages of other algorithms. It can only manage to reduce one or another disadvantage, improving the iteration speed or calculation accuracy. The detection of particulate matter across the world mainly focuses on inorganic molecules distributed in the air, rather than organic ones dissolved in the liquid media (He et al., 2018).

The following issues arise when particle matter in milk is detected: during the detection of organic molecules in a liquid medium, the medium may absorb light and affect the outcomes; in the total light scattering method, the first kind of Fredholm integral equation is a typical ill-posed problem; when calculating particle size, the algorithm exhibits delayed convergence, inaccurate inversion and poor noise resistance (Maguire et al., 2018).

This research suggests an enhanced MPGA based on the total light scattering approach to address these issues. It simplifies the objective function using Tikhonov smooth function (Wang and Sun, 2013) and reduces ill conditions of the inversion equation. Based on the extinction spectra calculated by known specific parameters, an inversion algorithm is built to generate a more precise particle size distribution. Apparent optical parameters (Fu and Sun, 2001; Yang et al., 2002; Fu and Sun, 2006; Ding et al., 2020; Wrana et al., 2020) are introduced based on total light scattering to solve the extinction coefficient and create an objective function. This effectively overcomes the influence of the light-absorbing medium. The study also achieves the particle size inversion of milk fat in medium of liquid absorption, addresses the typical ill-posed problem in the total light scattering method, and provides a reliable method for determining particle size of milk fat (Lienert et al., 2001; Wang et al., 2021; Zhou et al., 2021; Cai et al., 2022).

## Materials and methods

### Experimental materials

The size of the milk fat particles in this study, which satisfied the unimodal R-R distribution function, was 0.1 μm ∼10 μm . Ethanolamine, of which the refractive index was $m=1.454+1.59\times 10^{-5}i$ and could be classified as liquid-absorbing medium, was selected as the medium to dissolve milk fat particles. Anhydrous milk fat was dissolved in the ethanolamine solvent at a concentration of 6% to create milk fat simulated liquid. The liquid was then thoroughly churned using a JJ-1 precision timed mixer while being maintained at a constant 30 °C temperature. The prepared simulated liquid was then measured with a Malvern particle size analyzer. According to the analyzer, the size of the measured particle system was 5.075  μm (Ding et al., 2020).

### Design of optical experimental system

**FIGURE 1:**
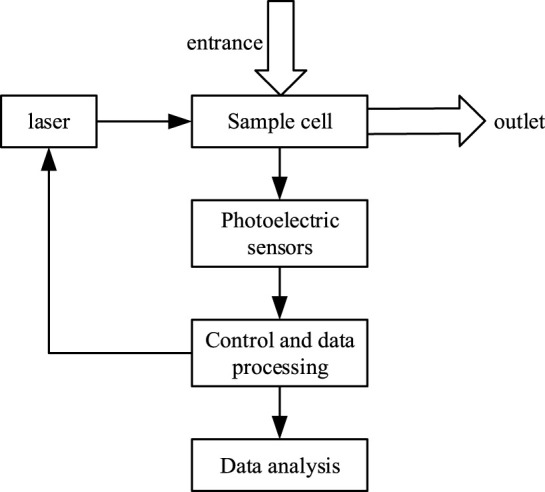
Schematic diagram of particle optical experimental system.

Figure 1 shows the schematic diagram of the optical experimental system. As can be seen from Figure 1, the whole system included photoelectric sensor, light source, data receiving unit and sample pool. Lasers of 0.3838, 0.4598, 0.5163, 0.6269 and 0.77 μm were selected as the light source (Ding et al., 2020). To improve accuracy, the study used quartz cuvette with low light consumption as the sample pool, the thickness of which was 12.33 mm. The light intensity of each wavelength was collected by the optical power detection device (optical power meter) (Ding et al., 2020).

### Measurement method

First, the ethanolamine medium was added into the sample pool. The measuring area was irradiated with a laser at a preset wavelength. Then, the scattered light intensity was collected by a photoelectric sensor positioned at a 90° angle. The collected signal was input to a computer for processing and was set as the incident light intensity. Then the simulated liquid containing milk fat particles was added to the sample pool in place of the original liquid. Using the same method above, the signal value was recorded as the scattered light intensity. To avoid errors, the ratio of the first result and the second result was processed and calculated by the computer as the extinction value (Wang, 2013). The excitation experiment was repeated for 20 times on the simulated liquid of milk fat particles with lasers of different wavelengths, and the average of the results was taken as the final light intensity (Cai et al., 2010; Wang, 2013; Wang and Sun, 2013; Ding et al., 2020).

### Basic concept and improvement of total light scattering method

The measurement of the particle size followed the Lambert-Beer law.

The monochromatic parallel lights’ incident intensity was $I_{0}$ and their wavelength was λ . When the lights were incident on the particle system, they was scattered and absorbed, and the transmitted intensity I would attenuate. The change can be expressed as (Cai et al., 2010):

−dI=IτdL (1)

Where: τ was the turbidity of the particle medium and L was the thickness of the medium to be measured. Assume that τ in the particle system was independent of the optical path *L*. In other words, the spatial distribution in the medium was disordered and uniform. Eq. 1 can be integrated along L :

$$-\int_{I_{0}}^{I}\frac{1}{I}dI=\int_{0}^{L}\tau dL$$ (2)

The relationship between the incident light intensity and the transmitted light intensity was changed into:

$$ln\frac{I\left(\lambda \right)}{I_{0}\left(\lambda \right)}=-\tau \left(\lambda \right)L$$ (3)

Where: $I\left(\lambda \right)/I_{0}\left(\lambda \right)$ was the extinction value with a wavelength of λ , τ(λ) was the turbidity with a wavelength of λ .

When the to-be-measured particles satisfy the condition of irrelevant single scattering and were all spherical particles with the size of D. In the single scattering system, the $N_{D}$ single particle’s extinction value was:

$$ln\frac{I\left(\lambda \right)}{I_{0}\left(\lambda \right)}=-\frac{\pi }{4}LN_{D}D^{2}Q_{ext}\left(\lambda ,m,D\right)$$ (4)

Where: $Q_{ext}\left(\lambda ,m,D\right)$ was the extinction coefficient, in which m was the complex refractive index of the relative medium. $Q_{ext}$ can be deduced based on Mie scattering theory: (Cai et al., 2010)

$$Q_{ext}=\frac{2}{\alpha^{2}}\sum_{n=1}^{\infty }\left(2n+1\right)Re\left(a_{n}+b_{n}\right)$$ (5)

Where: α=πD/λ was the size parameter of zero-dimensional particles; $a_{n}$ and $b_{n}$ were the Mie coefficients related to Bessel and Hankel functions.

$$a_{n}=\frac{\psi_{n}^{\prime }\left(m\alpha \right)\psi_{n}\left(\alpha \right)-m\psi_{n}\left(m\alpha \right)\psi_{n}^{\prime }\left(\alpha \right)}{\psi_{n}^{\prime }\left(m\alpha \right)\xi_{n}\left(\alpha \right)-m\psi_{n}\left(m\alpha \right)\xi_{n}^{\prime }\left(\alpha \right)}$$ (6)

$$b_{n}=\frac{m\psi_{n}^{\prime }\left(m\alpha \right)\psi_{n}\left(\alpha \right)-\psi_{n}\left(m\alpha \right)\psi_{n}^{\prime }\left(\alpha \right)}{m\psi_{n}^{\prime }\left(m\alpha \right)\xi_{n}\left(\alpha \right)-\psi_{n}\left(m\alpha \right)\xi_{n}^{\prime }\left(\alpha \right)}$$ (7)

$$\psi_{n}\left(\alpha \right)=\sqrt{\frac{\pi \alpha }{2}}J_{n+1/2}\left(\alpha \right)$$ (8)

$$\xi_{n}\left(\alpha \right)=\sqrt{\frac{\pi \alpha }{2}}H_{n+1/2}^{2}\left(\alpha \right)$$ (9)

In Eqs 8, 9, $J_{n+1/2}$ and $H_{n+1/2}$ were the Bessel function of half-integer order and the Hankel function of first kind, respectively. $m=m_{r}+m_{i}i$ was the complex refractive index of the particles relative to their surrounding medium. The sum of infinite series was considered when we calculated the extinction coefficients using Mie scattering theory. The sum upper limit $N_{stop}$ was usually derived by the empirical formula of Wiscombe (Wang, 2013; Wang and Sun, 2013):

$$N_{stop}=\{\begin{array}{c} \alpha +4\alpha^{1/3}+1,0.02\leq \alpha \leq 8\\ \alpha +4.05\alpha^{1/3}+2,8\leq \alpha \leq 4200\\ \alpha +4\alpha^{1/3}+2,4200\leq \alpha \leq 20000 \end{array}$$ (10)

The calculation formula of the extinction coefficient $Q_{ext}$ in Mie scattering theory was:

$$Q_{ext}=\frac{2}{\alpha^{2}}\sum_{n=1}^{N_{stop}}\left(2n+1\right)Re\left(a_{n}+b_{n}\right)$$ (11)

In measurement, the particle system was a dispersion with multiple scattered matters rather than a monodisperse system. Thus, when we had the wavelengths, the extinction value of spherical particles was calculated with the following equation:

$$ln\frac{I\left(\lambda \right)}{I_{0}\left(\lambda \right)}=-\frac{\pi }{4}L\int_{D_{min}}^{D_{max}}Q_{ext}\left(\lambda ,m,D\right)N\left(D\right)D^{2}dD$$ (12)

Where: $D_{max}$ and $D_{min}$ were the maximum and minimum values that meet the particle size distribution in the system; N(D) was the number of particles in [D+dD] . Volume distribution was used for calculation, because quantity distribution was relatively complex:

$$ln\frac{I\left(\lambda \right)}{I_{0}\left(\lambda \right)}=-\frac{3}{2}LN_{D}\int_{D_{min}}^{D_{max}}\frac{Q_{ext}\left(\lambda ,m,D\right)}{D}f\left(D\right)dD$$ (13)

Where: $N_{D}$ was the total number of to-be-measured particles; f(D) was the volume frequency distribution function of the particle system. This formula was the Fredholm integral equation of the first kind. As it is difficult to calculate directly, the equation can be further discretized, as shown in Eq. 14:

$$ln\frac{I\left(\lambda \right)}{I_{0}\left(\lambda \right)}=-\frac{3}{2}LN_{D}\sum_{j=1}^{S}c_{j}\frac{Q_{ext}\left(\lambda ,m,\tilde{D_{j}}\right)}{\tilde{D_{j}}}f\left(D_{j}\right)$$ (14)

Where: S denoted the grading number of particle size in $\left[D_{min},D_{max}\right]$ ; $c_{j}$ was the numerical integration coefficient; $f_{j}\left({\tilde{D}}_{j}\right)=\overset{j+1}{\underset{j}{\int }}f\left(D\right)d\left(D\right)$ ; and ${\tilde{D}}_{j}$ represented the midpoint of subinterval $\left[D_{j},D_{j+1}\right]$ . There were S types of particle size distribution to be solved, and they were measured using multiple wavelengths. As a result, the linear equation set was obtained (Wang, 2013; Wang and Sun, 2013):

E=Af (15)

Where: $E=\left[ln\left(I_{1}/I_{10}\right);\cdots ln\left(I_{U}/I_{U0}\right)\right]$ ; U was the wavelength; and $A=\left[A_{ij}\right]$ indicated the U×S weight matrix, with all the elements expressed as: $A_{ij}=-3C_{j}LN_{D}Q_{ext}\left(\lambda_{i},m,{\tilde{D}}_{j}\right)/\left(2{\tilde{D}}_{J}\right),i=1,\cdots ,U,j=1,\cdots ,S$ ; $f=\left[f_{1}\left({\tilde{D}}_{1}\right),\cdots ,f_{S}\left({\tilde{D}}_{S}\right)\right]$ .

Inversion of particle size distribution by total light scattering method used algorithms to inverse particle size, with given extinction values of incident light wavelengths. Based on whether the distribution function was preset in data processing, the inversion can be divided into dependent algorithm and independent algorithm. After optical measurement of the simulated liquid of milk fat particle, the extinction value was obtained. Then, total light scattering method was used to inverse particle size. (The experiment and simulation results were obtained by dependent algorithm).

Dependent algorithm, also known as function restriction method, required presetting a distribution function during the calculation. Its concept was simple. The measured extinction values of multiple incident light wavelengths need to be substituted into the preset distribution function to solve the characteristic parameters of the function. This method was widely used for its simple steps. The specific steps in this paper were as follows:

The distribution function of the to-be-measured particle system was preset as follows:

$$f\left(D\right)=f\left(D,D_{1},D_{2}\right)$$ (16)

Where: $D_{1}$ and $D_{2}$ were the characteristic parameters of the distribution function f(D) .

In inversion, Eq. 16 was substituted into Eq. 14 by referring to extinction values:

$$ln\frac{I{\left(\lambda \right)}_{i}}{I_{0}{\left(\lambda \right)}_{i}}=-\frac{3}{2}LN_{D}\sum_{j=1}^{S}c_{j}\frac{Q_{ext}\left(\lambda_{i},m,{\tilde{D}}_{j}\right)}{{\tilde{D}}_{j}}f_{j}\left({\tilde{D}}_{j},D_{1},D_{2}\right)$$ (17)

Where: i indicated the measured wavelength $\lambda_{i}$ . The objective function was:

$$\text{OBE}={\left|\frac{ln{\left(I/I_{0}\right)}_{i}}{ln{\left(I/I_{0}\right)}_{k}}-\frac{-\frac{3}{2}LN_{D}\sum_{j=1}^{S}c_{j}\frac{Q_{ext}\left(\lambda_{i},m,{\tilde{D}}_{j}\right)}{{\tilde{D}}_{j}}f_{j}\left({\tilde{D}}_{j},D_{1},D_{2}\right)}{-\frac{3}{2}LN_{D}\sum_{j=1}^{S}c_{j}\frac{Q_{ext}\left(\lambda_{k},m,{\tilde{D}}_{j}\right)}{{\tilde{D}}_{j}}f_{j}\left({\tilde{D}}_{j},D_{1},D_{2}\right)}\right|}^{2},i\neq k$$ (18)

Thus, the problem of inversing to-be-measured particle size distribution transformed into solving parameters $D_{1}$ and $D_{2}$ corresponding to the minimum objective function. By substituting parameters $D_{1}$ and $D_{2}$ into Eq. 19, the Sauter average particle size was obtained and compared with the actual particle size (Wang, 2013; Wang and Sun, 2013).

$$D_{32}=\frac{\int_{D_{min}}^{D_{max}}D^{3}f\left(D\right)dD}{\int_{D_{min}}^{D_{max}}D^{2}f\left(D\right)dD}$$ (19)

Where: f(D) was the distribution function of the particle size; D denoted the single particle size; $D_{max}$ and $D_{min}$ represented the maximum and minimum particle sizes in the particle system.

Based on the method of total light scattering, Tikhonov regularization was introduced into the objective function. The equation was:

$$min=\|E-Af{\|}^{2}+\alpha \|f{\|}^{2}$$ (20)

Where: α denoted the regularization parameter which adjusted the relative weight between the residual $E-Af^{2}$ and the regularized $f^{2}$ . The Tikhonov smooth objective function was:

$$\varphi \left(f\right)=min\left\{\begin{array}{c} \overset{s}{\underset{i=1}{\sum }}{\left\{ln{\left(\frac{I}{I_{0}}\right)}_{\lambda_{i}}-\left[-\frac{3}{2}LN_{D}\sum_{j=1}^{N}c_{j}\frac{Q_{ext}\left(\lambda_{i},m,D_{j}\right)}{D_{j}}f\left(D_{j}\right)\right]\right\}}^{2}\\ +\alpha \sum_{j=1}^{N}{\left[f\left(D_{j}\right)\right]}^{2} \end{array}\right\}$$ (21)

Where: when calculating the extinction value, the apparent optical scattering principle was used, so light absorption character of the liquid medium should be considered. The regularization parameter was solved by the L curve method (Yang, 2016; Zhou et al., 20212021), in which the maximum curvature point was the most appropriate regularization parameter. Let $\rho =lgb-Ax_{2}^{\alpha }$ , $\theta =lgx_{2}^{\alpha }$ , then the curvature can be defined as (Yang, 2016; Wang et al., 2019; Zhou et al., 20212021):

$$K\left(\alpha \right)=\frac{\rho^{\prime }\theta^{\prime \prime }-\rho^{\prime \prime }\theta^{\prime }}{{\left[{\left(\rho^{\prime }\right)}^{2}+{\left(\theta^{\prime }\right)}^{2}\right]}^{\frac{3}{2}}}$$ (22)

Where: The α , which corresponded to the maximum value of K(α) , was the desired regularization parameter. Thus, the problem of inversing particle size distribution was transformed to finding the minimum value of the objective function.

After the objective function was constructed by Tikhonov (Yang, 2016; Wang et al., 2019; Zhou et al., 20212021) regularization, MPGA (Guo et al., 2020; Shi et al., 2021; Yang et al., 2022) was introduced to find the minimum value.

### Solving the objective function minimum value with MPGA

1) Taking the unimodal R-R distribution function as an example, the first step was to determine $\bar{D}$ and K, the characteristic parameter values of the R-R distribution function. Then, n initial populations were randomly generated. Each population contained m individuals. Each individual, which was coded in binary to form a gene coding of a chromosome, represented different set of $\bar{\;D}$ and K, which were the characteristic parameter values of a R-R distribution function.
2) The objective fitness function was determined. The function in this paper was $\text{y}=E-Af^{2}$ and $\text{y}=E-Af^{2}+\alpha f^{2}$ .
3) The fitness of each individual in each community was calculated using the substituted individuals, and the findings were rated within each population from high to poor.
4) Excellent individuals were moved from each subpopulation to adjacent subpopulations to replace low-fitness individuals.
5) Selection operation was carried out. To scale the fitness value of individuals in each sub-population, Roulette Wheel Selection was adopted. The chance of elimination would increase as the fitness rating decreased. Then the individual was assessed to see if it met the optimization criteria. If it was qualified, the results would be decoded and exported to obtain the optimal solution and the iteration stopped; Otherwise, the next step would be taken.
6) The fitness function values were compared to determine the regenerated individuals of the population. Excellent individuals were picked and reproduced manually and added to the elite population to be saved. It was also necessary to determine whether they were repeated. If so, the repeated one would be eliminated and the selection operation would start.
7) Crossover and mutation were carried out based on the randomly generated probabilities of each subpopulation. This was to obtain next generation of populations and individuals.
8) A new generation of populations were generated by crossover and mutation, and returned to step (3).
9) Iteration ceased and the results were exported when the algorithm reached the predetermined accuracy. Otherwise, crossover and mutation would continue in step (5) to produce new individuals and populations. The least preserver algebra in the elite population was determined until the preset iterative cutoff value was met.

**FIGURE 2:**
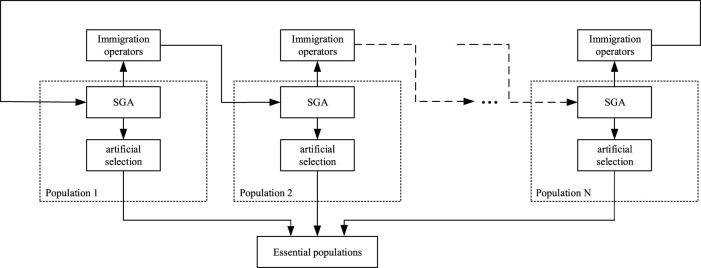
MPGA structure diagram.

Figure 2 shows the MPGA structure diagram. Each population was an independent evolution of standard genetic algorithm (SGA), in which single-point crossover and site mutation were used for global search, and roulette for selection (Bouvier et al., 2019).

### Data analysis

Excel 2016 was used to process the experiment’s preliminary statistical data. Data and graphics were analyzed and processed with Matlab 2016a, including the calculation of apparent extinction coefficient (Fu and Sun, 2001; Yang et al., 2002; Fu and Sun, 2006; Ding et al., 2020; Wrana et al., 2020), regularization parameter and Mie scattering coefficient. Matlab 2016a was used to inverse different algorithms for calculating particle size distribution. Sauter average particle size $D_{32}$ (Han et al., 2021; Romanov et al., 2021; Tolosana-Moranchel et al., 2021) was applied to assess the accuracy of the measured samples.

## Results and analysis

### Model establishment and analysis of the simulation experiment

First, numerous simulation experiments were used to verify the accuracy and reliability of the algorithm. The commonly used R-R function was adopted as the particle size distribution function for the inversion with a dependent algorithm (Xu and Nieto-Vesperinas, 2019; Liu et al., 2021; Li et al., 2022). In other words, the characteristic parameters in the known equation set was determined with optimal solution. The volume frequency distribution function under unimodal and bimodal conditions was as follows (Cai et al., 2010):

$$f_{R\text{R}-\text{s}}\left(D\right)=\frac{K}{\bar{D}}\cdot {\left(\frac{D}{\bar{D}}\right)}^{K-1}\cdot e^{\left[-{\left(\frac{D}{\bar{D}}\right)}^{K}\right]}$$ (23)

$$f_{RR-b}\left(D\right)=n\left[\frac{K_{1}}{{\bar{D}}_{1}}\cdot {\left(\frac{D}{{\bar{D}}_{1}}\right)}^{K_{1}-1}\cdot e^{\left[-{\left(\frac{D}{{\bar{D}}_{1}}\right)}^{K_{1}}\right]}\right]+\left(1-n\right)\left[\frac{K_{2}}{{\bar{D}}_{2}}\cdot {\left(\frac{D}{{\bar{D}}_{2}}\right)}^{K_{2}-1}\cdot e^{\left[-{\left(\frac{D}{{\bar{D}}_{2}}\right)}^{K_{2}}\right]}\right]$$ (24)

Where: RR−s and RR−b represented unimodal and bimodal RR distributions, respectively. Moreover, $\bar{D}$ , k , ${\bar{D}}_{1}$ , $k_{1}$ , ${\bar{D}}_{2}$ , $k_{2}$ were the characteristic parameters, 0≤n≤1 .

The complex refractive index was set at m=1.33+0.01i . The simulation extinction values of incident wavelengths used 0.3838, 0.4598, 0.5163, 0.6269 and 0.77  μm . In SGA, the population size, probability of selection, and mutation probability was set at 50, 0.7, and 0.05, respectively. The iteration times of unimodal and bimodal distribution functions were set at 200 and 300, respectively. In MPGA, the initial population number was set at 10, population size 50, selection probability [0.7, 0.9], and mutation probability [0.001.0.05]. The iteration times were the same as in the SGA. The approximate range of distribution parameters following RR distribution was $\text{R}-\text{R}:1<\text{k}<9;1<\bar{\text{D}}<9$ , which was inferred from the particle size range of 0.1∼10 μm . The inversion error was defined as follows (Doicu et al., 2019; Shcherbakov et al., 2019; Zhang et al., 2019):

$$\text{δ}=\frac{\sqrt{\sum_{j=1}^{S}{\left[f\left(D_{j}\right)-F\left(D_{j}\right)\right]}^{2}}}{\sqrt{\sum_{j=1}^{S}{\left[f\left(D_{j}\right)\right]}^{2}}}$$ (25)

First, the research verified the effect of regularization and non-regularization objective functions on the reconstruction of particle size distribution. It compared the results with 5% noise or without noise with MPGA, respectively. Then, the least squares error formula and the LS error with Tikhonov smooth function constraint were used as the objective fitness functions. As a result, the following regularization settings were specified for the latter:

The regularization parameters without noise and with 5% noise were $9.329\times 10^{-6}$ and $6.142\times 10^{-4}$ under unimodal distribution function, respectively. As for bimodal distribution function, the parameters were $5.231\times 10^{-5}$ and 0.0028, respectively.

**FIGURE 3:**
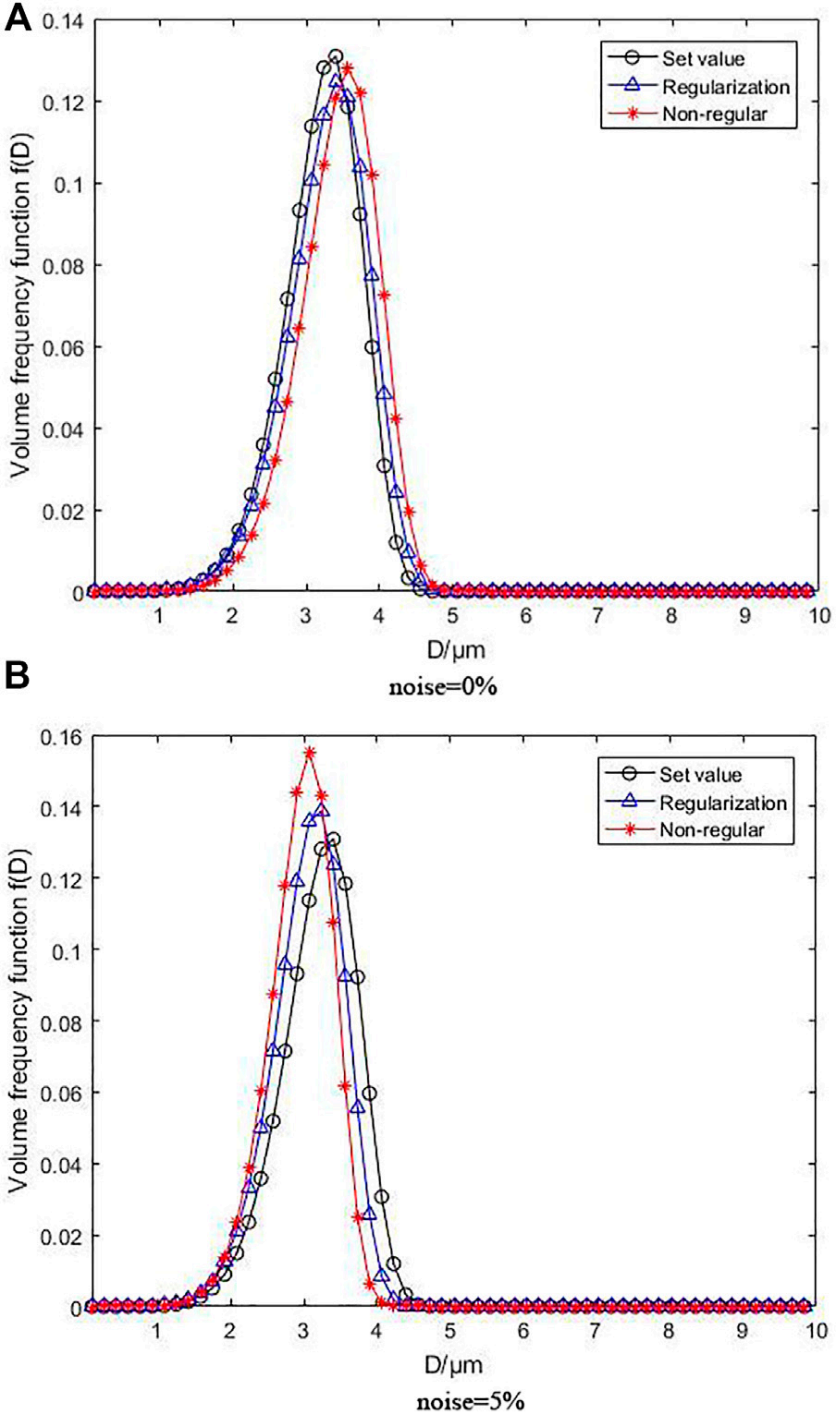
Inversion results following unimodal R-R distribution without noise and with 5% noise.

Figure 3 depicts the inversion results for the unimodal R-R distribution, where Figure 3A under the condition of without noise and Figure 3B under the condition of with 5% noise. When no noise is added, the peak position and height of the volume frequency distribution function curves generated by MPGA inversion based on regularization and non-regularization objective functions are very close to the set values, particularly when the peak position of the regularization basically matches the preset curve, despite the variation of the peak height. However, the identification of particle size distribution is unaffected, although the curve distribution of non-regularization moves smoothly. Despite the fact that the peak position is deviated, resulting in judgment errors in the particle size distribution, the total effect is within the error range. When 5% noise is added, the regularization curve modifications are not apparent, which is consistent with the preset distribution curve. Although the peak height varied slightly, it is still within an acceptable range, demonstrating that the inversion algorithm with regularization is strongly noise resistant. The function curve with non-regularization has a great peak position and height offset as the noise increases, while the reconstruction effect is low, showing that the noise resistance of the inversion algorithm without regularization is inadequate.

**FIGURE 4:**
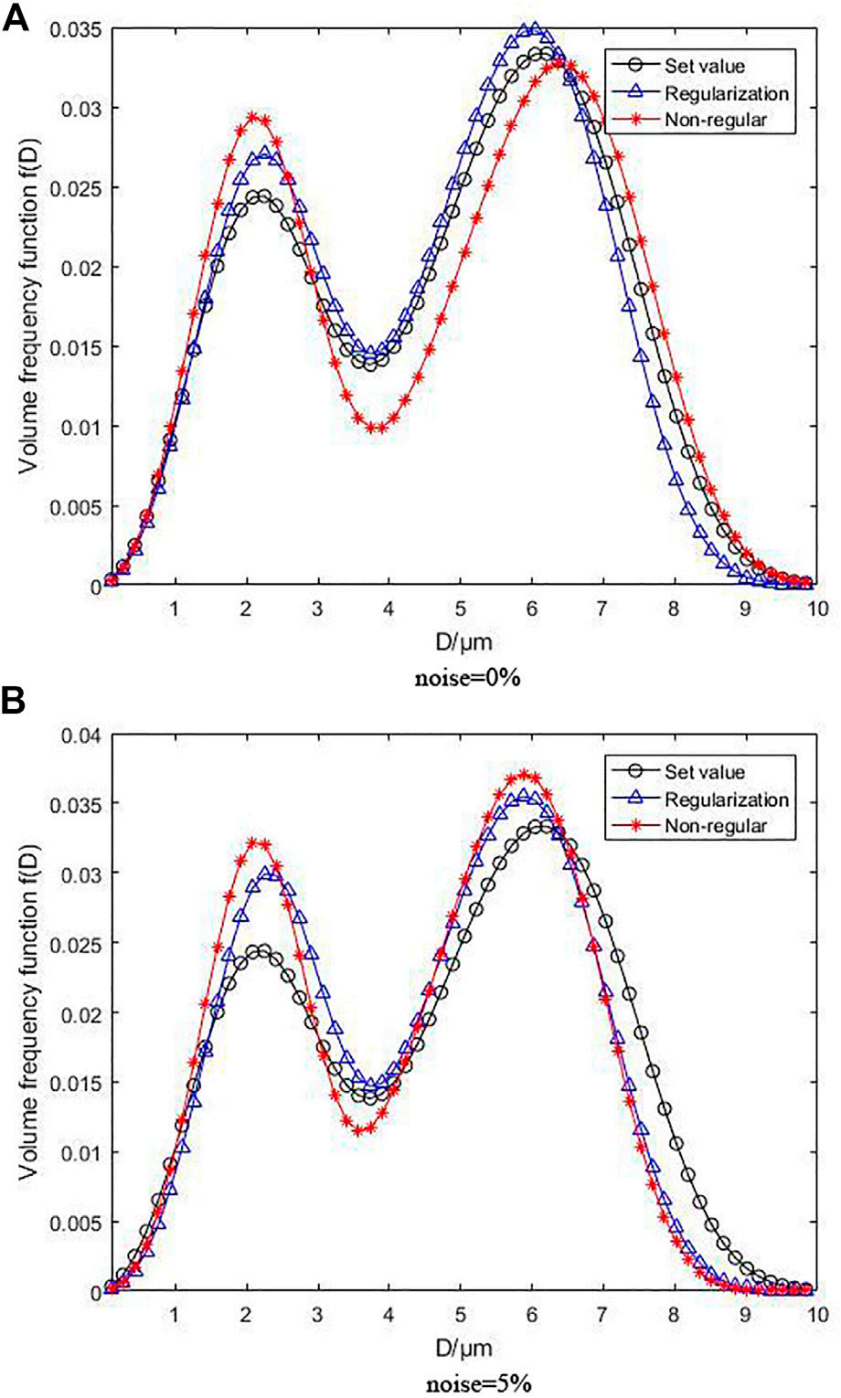
Inversion results following bimodal R-R distribution without noise and with 5% noise.

Figure 4 illustrates the inversion results following for the bimodal R-R distribution, where Figure 4A under the condition of without noise and Figure 4B under the condition of with 5% noise. As can be observed, the volume frequency distribution function curves produced by MPGA inversion based on regularization objective function are highly consistent with one another in the absence of noise. The error is acceptable despite slightly higher peaks. The peak positions are basically identical, which does not affect the particle size distribution identification. However, the overall curve deviation of non-regularization is quite large, and the height deviation of the first peak and the position deviation of the second peak are obvious. Additionally, the inversion impact was reduced due to the complexity of the bimodal distribution function, demonstrating that an inversion algorithm with regularization outperforms the one with non-regularization in complex function inversion. Even when 5% noise is added, the overall effect of the regularization curve is still better than that of non-regularization. As the noise rises, the peak heights of the inversion curve for both objective functions grow, indicating that the overall inversion impact is proportional to the bimodal distribution function’s complexity. However, the curve produced by the inversion algorithm with regularization is closer to the preset distribution curve, suggesting that the inversion algorithm with regularization is still more noise-resistant than that with non-regularization even under complex bimodal distribution function.

**FIGURE 5:**
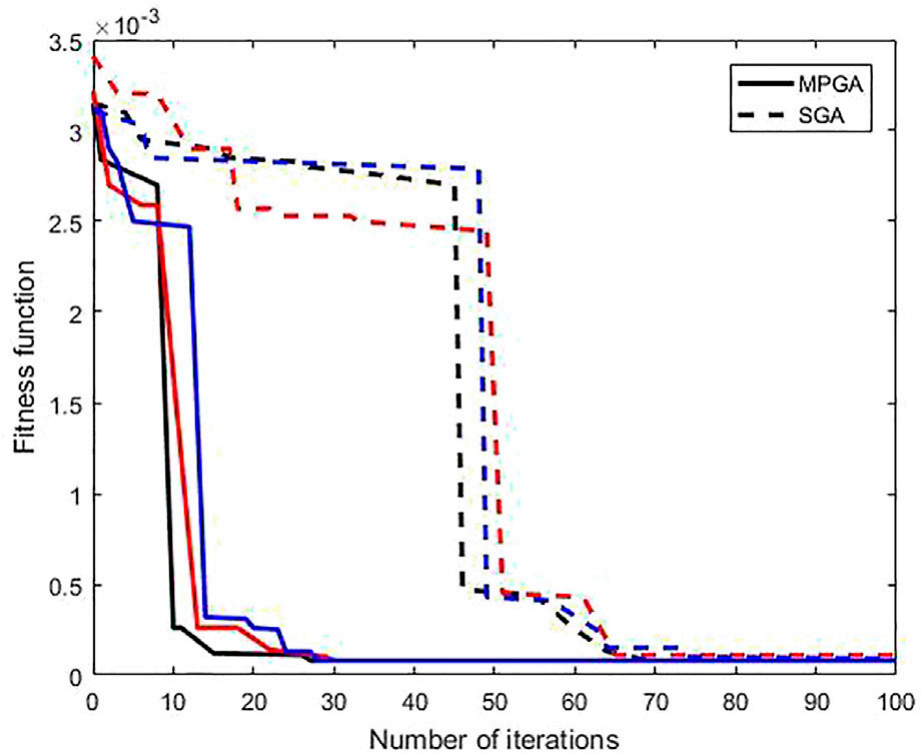
Change rule of the fitness function value varying with iteration times with SGA and MPGA.

Next, the research then calculated the inversion of the particle size distribution with MPGA and SGA with effects compared to verify the reliability of improved genetic algorithm based MPGA. Figure 5 shows the changing rules of the fitness function value as it varies with iteration times under the unimodal distribution function for SGA and MPGA, where the fitness function is regularization objective function. Figure 5 shows that iteration results using SGA stabilize after 65 iterations, despite the fact that the findings are not unique. Comparatively, MPGA iteration outcomes stabilized after 25 rounds, yielding consistent final findings.

**FIGURE 6:**
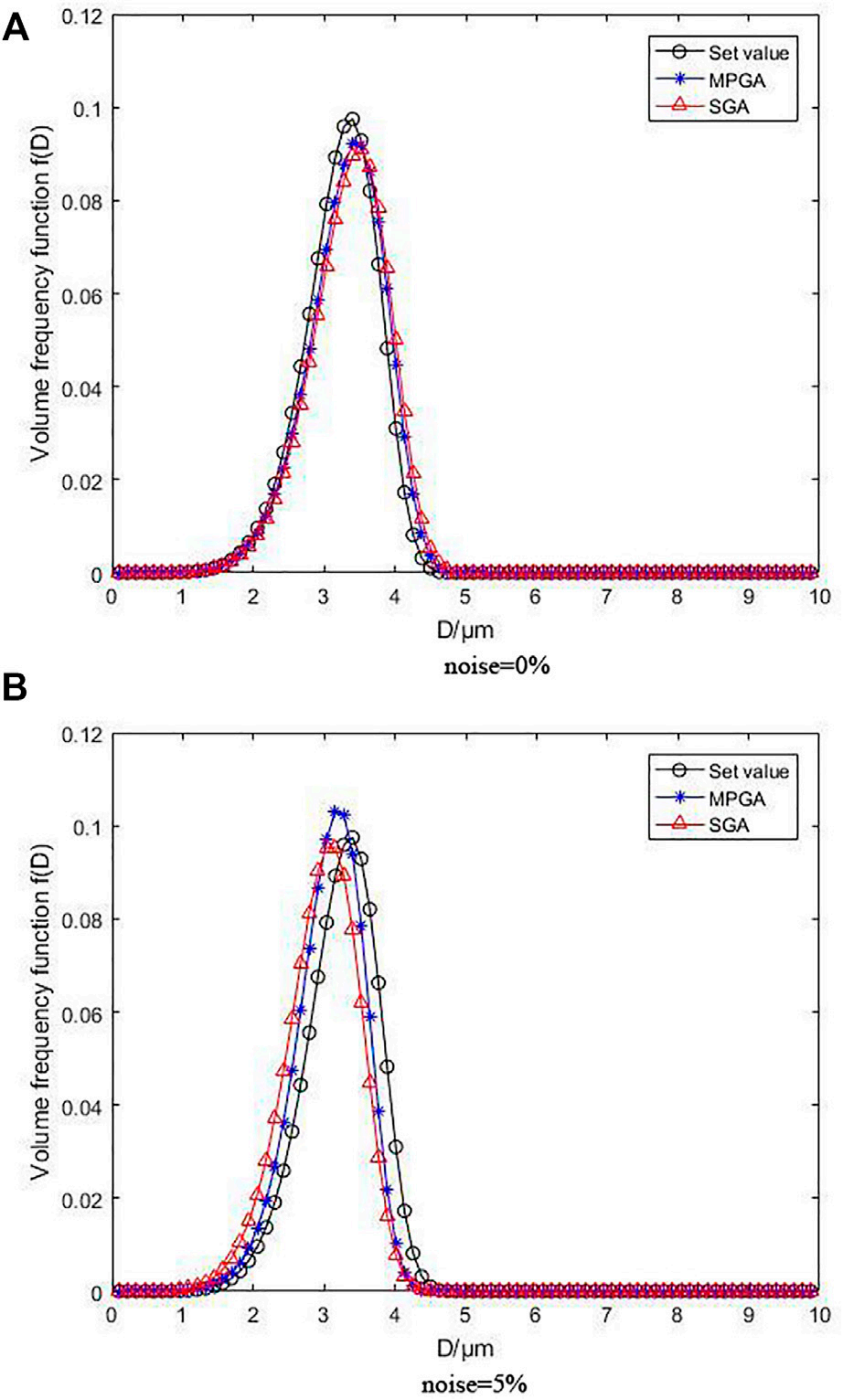
Inversion results following unimodal distribution with different algorithms.

**FIGURE 7:**
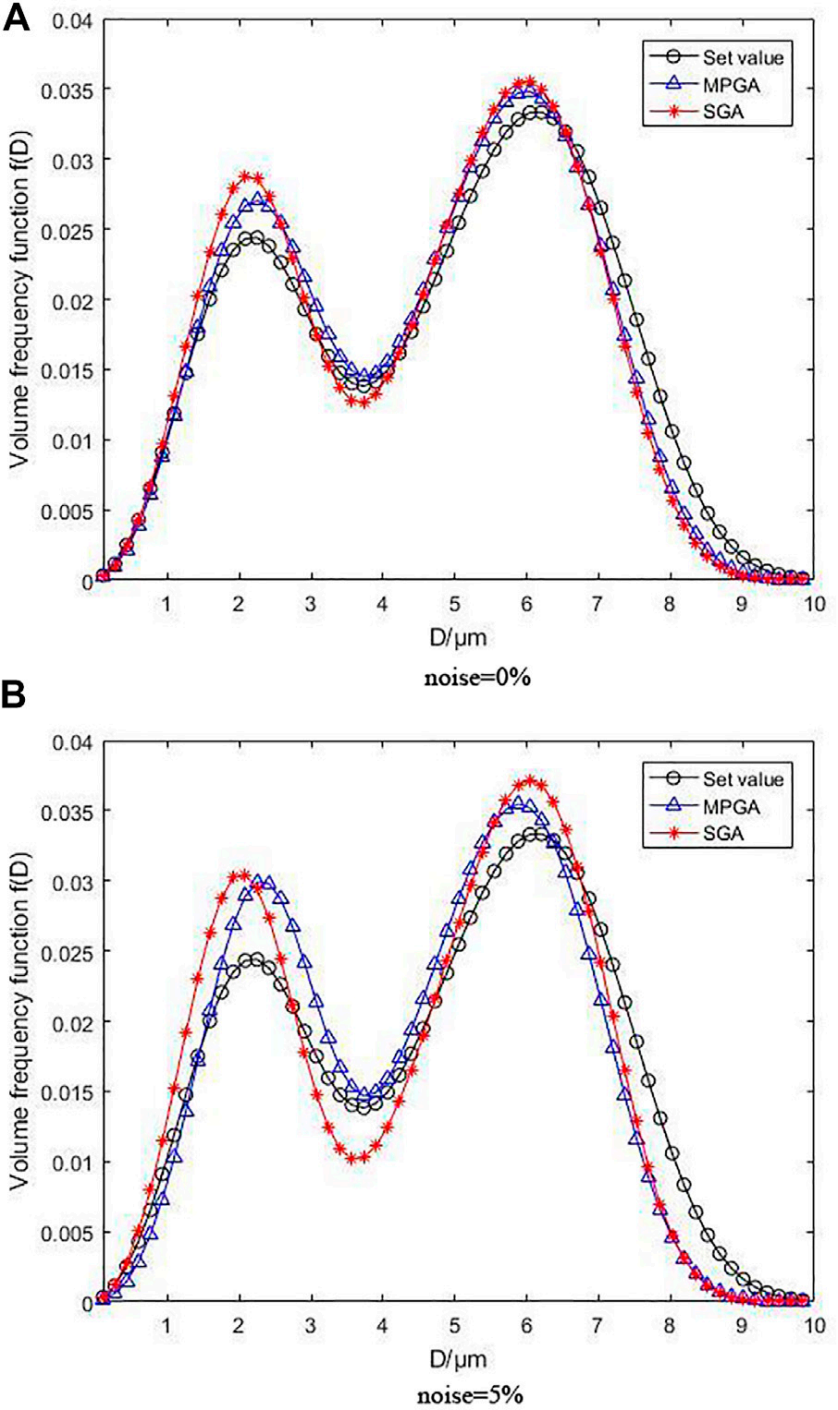
Inversion results following bimodal distribution with different algorithms.

Figures 6, 7 show the volume frequency distribution function curve calculated based on the inversion of particle size distribution after unimodal and bimodal R-R functions with MPGA and SGA, where Figures 6A, 7A under the condition of without noise and Figures 6B, 7B under the condition of with 5% noise. Without noise, the inversion results of both algorithms for unimodal and bimodal functions agree with the predetermined values, and the peak position and height could be rebuilt effectively. Among them, the early peak heights of the bimodal distribution function deviates to a certain degree, however, the peak height deviates further with SGA due to the immature convergence of SGA in the complex objective function situation. The immature convergence of SGA exacerbated by the addition of 5 percent noise, and both the unimodal and bimodal functions exhibit substantial deviations. The inversion effect of MPGA is slightly superior to that of SGA. Although the peak location and height vary, they are still acceptable and the overall effect is superior to that of SGA, indicating that the anti-noise ability of MPGA is outperforms the capability of SGA.

### Experimental results and analysis

Considering the light absorption of liquid medium, milk fat particles were employed as the research object in actual measurements to further validate the paper’s algorithm. Table 1 displays the extinction levels measured at various wavelengths determined by the mean value of 20 experimental data groups.

**TABLE 1:** Extinction values measured at different wavelengths.

	0.3838 μm	0.4598 μm	0.5163 μm	0.6269 μm	0.77 μm
Average value	0.5137	0.5101	0.5127	0.4964	0.4851

Table 2 Displays the inversion data obtained from five inversion tests with SGA and MPGA. According to Table 1, the k values of both algorithms are relatively substantial, indicating that the particle size distribution of the simulation solution of milk fat in the experiment is relatively concentrated and consistent with narrow band distribution. The results of the inversion indicate that the final average values of the two algorithms are quite similar. In contrast, in many trials, the final iteration’s SGA findings are variable, whereas MPGA results are consistent. This demonstrates that MPGA is more robust and effective at optimizing systems and addressing complicated issues.

**TABLE 2:** Comparison of inversion results between SGA and MPGA.

Serial number	SGA( $\bar{D}$ ,k)	MPGA( $\bar{D}$ ,k)
01	(5.0202.17.7576)	(5.0793.17.3047)
02	(4.9958.17.7161)	(5.0793.17.3047)
03	(5.0049.17.7135)	(5.0793.17.3047)
04	(4.9928.17.6846)	(5.0793.17.3047)
05	(4.9704.17.7103)	(5.0793.17.3047)
Average value	(4.9968.17.7164)	(5.0793.17.3047)

**FIGURE 8:**
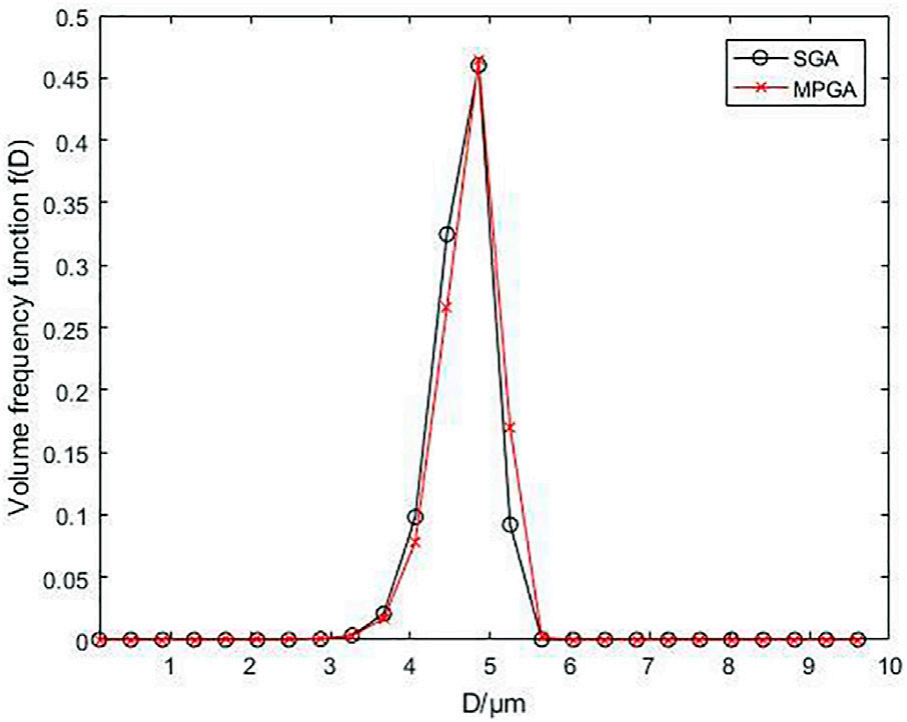
Comparison of volume frequency distribution maps obtained from inversion with SGA and MPGA.

Figure 8 illustrates the particle size results obtained with SGA and MPGA. In Figure 8, the Sauter average particle sizes $D_{32}$ are 4.8206  μm and 4.8962  μm , respectively, while the relative errors are -5.01% and -3.52%, respectively, meeting the national standard of ±8%.

## Discussion

### Comparative analysis of the inversion results of different objective functions

The least square error is frequently used as the objective function in particle size inversion calculations. In their book (Cai et al., 2010), Cai et al. elaborated on the solution of objective functions and emphasized that the least squares error is used as the objective function for inversion calculations. In practical calculation, however, the extinction coefficient determines the linear equation of the extinction spectrum, primarily because the linear equation resulting from the highly ill-conditioned coefficient matrix is unstable as a result of the glitches generated during the initial calculation process. If we directly solve the least-squares solution as an objective fitness function, the calculation results may not be unified with a reduced level of precision. Therefore, the introduction of Tikhonov regularization into the creation of objective functions can effectively solve the problem of ill solutions. The main goal of Tikhonov regularization is to modify the relative weight between residual and regularization terms and to reduce the ill-condition of the objective function by the introduction of smooth constraints, hence optimizing the stability of the function to be measured. The approach changes the problem into a solution for least-squares problems with penalty factors. By introducing regularization parameters, the literature (Yang, 2016; Wang et al., 2019; Zhou et al., 20212021) efficiently addresses the problem of coefficient matrix pathology encountered during the development of objective functions.

Table 3 compares the characteristic parameters and inversion errors (using MPGA for inversion) for unimodal and bimodal distribution functions calculated using the inversion algorithm with regularization, non-regularization, and 5% noise. As shown in Table 3, the inversion errors of the objective function built by regularization and non-regularization under the unimodal R-R distribution function are 0.0402 and 0.0974, respectively, without noise, and 0.0799 and 0.1450, respectively, with 5% noise. Non-regularization under the bimodal R-R distribution function yields the following results: the inversion errors are 0.0539 and 0.1085 without noise, and 0.0917 and 0.1473, respectively, with 5% noise.

**TABLE 3:** Comparison of the characteristic parameters and the inversion errors under unimodal and bimodal distribution functions.

Distribution function	Objective function	Noise (%)	Inversion value	Inversion error
Unimodal R-R distribution (3.5, 7.55)	Regularization	0	(3.5903.7.3387)	0.0402
		5	(3.3278.7.6257)	0.0799
	Non-regularization	0	(3.7278.7.8257)	0.0974
		5	(3.2103.8.2039)	0.1450
(2.5.3.0.6.5.5.0.0.3) Bimodal R-R distribution (2.5, 3.0, 6.5, 5.0, 0.3)	Regularization	0	(2.5468.3.1385.6.3201.5.2904.0.3270)	0.0539
		5	(2.5832.3.4374.6.2069.5.3846.0.3385)	0.0917
	Non-regularization	0	(2.4463.3.2053.6.7364.5.4334.0.3432)	0.1085
		5	(2.4136.3.5233.6.1804.5.6306.0.3403)	0.1473

The simulation experiment compares the inversion errors under two different objective functions. By comparing the regularization and non-regularization objective functions, it was discovered that the inversion error with the regularization objective function was significantly smaller than that with the non-regularization objective function, regardless of whether the distribution functions were unimodal or bimodal. The regularization inversion error with 5% noise was even lower than the non-regularization inversion error without noise, indicating that the regularization had a positive effect on alleviating the ill condition of the discrete complex coefficient matrix, enhancing the accuracy of particle size distribution inversion results, and effectively reducing the complexity of the objective function and enhancing the stability. In addition, research data (Wang, 2013; Zhang, 2015; Zhao et al., 2015) indicate that the inversion of the regularization goal function yields excellent results.

### Comparative analysis of different inversion algorithms

This work compares the inversion outcomes of SGA and MPGA by calculating the inversion of the particle size of milk fat. We initially examined the iteration times. MPGA times were significantly less than those of SGA because MPGA utilised multiple populations simultaneously in the initial population setting, and the populations were both independent and interconnected. In addition, the optimal individuals from each population were preserved in the elite population for repeat selection and to avoid loss, thereby drastically lowering the number of iterations required to locate the optimal individuals. However, SGA only had one population per iteration for optimal individual selection, which was insufficient to assure loss and repeated selection. Through MPGA, the literature (Yang et al., 2022) significantly enhances the algorithm’s speed. In addition, MPGA’s final iteration findings were consistent, indicating that MPGA’s target parameter search was more accurate and exhaustive. It was mostly owing to the fact that the control parameters in optimal individual selection were distinct. In SGA, the crossover and mutation probabilities were fixed. The crossover and mutation operations determined the global and local search capabilities of the algorithm, while the probability setting influenced the algorithm’s overall search capability. The optimization results for various selections differed considerably. MPGA made up for SGA’s deficiency by randomly generating probability parameters within the specified range to distinguish the crossover and mutation probabilities for each subpopulation. In the meantime, populations evolved synchronously, covering both global and local searches of the algorithm. The problem of inconsistent experimental results is solved by MPGA in the literature (Guo et al., 2020; Shi et al., 2021).

Table 4 compares the characteristic characteristics and inversion errors (using regularization goal function for inversion) of particle size distribution functions produced by MPGA and SGA inversion without noise and with 5% noise. According to Table 3, the inversion results of MPGA and SGA under unimodal R-R distribution function are as follows: the inversion errors are 0.0402 and 0.0563, respectively, without noise; 0.0799 and 0.1158, respectively, with 5% noise; and under bimodal R-R distribution function, the inversion errors are 0.0539 and 0.0665, respectively, without noise; and 0.0917 and 0.1384, respectively, with 5% noise.

**TABLE 4:** Comparison of characteristic parameters and inversion errors of particle size distribution under unimodal and bimodal distribution functions.

Distribution function	Inversion algorithm	Noise (%)	Inversion value	Inversion error
Unimodal R-R distribution (3.5, 7.55)	MPGA	0	(3.5903.7.3387)	0.0402
		5	(3.3278.7.6257)	0.0799
	SGA	0	(3.6294.7.2882)	0.0563
		5	(3.2302.6.8374)	0.1158
(2.5.3.0.6.5.5.0.0.3) Bimodal R-R distribution (2.5, 3.0, 6.5, 5.0, 0.3)	MPGA	0	(2.5468.3.1385.6.3201.5.2904.0.3270)	0.0539
		5	(2.5832.3.4374.6.2069.5.3846.0.3385)	0.0917
	SGA	0	(2.4427.3.1973.6.2949.5.4367.0.3332)	0.0665
		5	(2.3463.3.1833.6.3234.5.8306.0.3453)	0.1384

According to the analysis, MPGA has better inversion effects than SGA, indicating that MPGA has stronger anti-noise capabilities. The inversion error comparison suggests that MPGA reconstruction is more precise than SGA reconstruction. Errors in SGA reconstruction could be decreased further, but doing so would require additional iterations and increase computation costs. SGA requires more search into random points, but MPGA effectively eliminates this drawback. Based on the outcome of this single operation, MPGA was able to better match the specified particle size distribution with high reconstruction accuracy under the same conditions, resolving the premature convergence of SGA. MPGA has faster iteration speed and higher inversion accuracy than SGA, hence avoiding the issue of SGA’s premature convergence.

## Conclusion

1) The combination of MPGA and Tikhonov smooth function supports particle size inversion calculations with the total light scattering method in an efficient manner. Due to their high dependability and stability, both functions reconstruct the particle size distribution in a liquid absorption medium.
2) Integrating Tikhonov regularization and apparent optical parameters into the construction of the objective function can effectively resolve the high ill condition of the objective function caused by the first type of Fredholm integral equation with the total light scattering method, as well as reduce the medium’s light absorption.
3) Compared to conventional genetic algorithms, the approach suggested in this study achieves a detection error of -3.52% with a greater detection accuracy, thereby providing a dependable method for milk molecular identification. The algorithm is ubiquitous and applicable to other domains as well.

## Data Availability

The original contributions presented in the study are included in the article/Supplementary Material, further inquiries can be directed to the corresponding author.
